# Detection of Quantitative Trait Loci (QTL) Associated with the Fruit Morphology of Tomato

**DOI:** 10.3390/genes11101117

**Published:** 2020-09-24

**Authors:** Pragya Adhikari, James McNellie, Dilip R. Panthee

**Affiliations:** 1 Department of Crop Sciences, University of Illinois at Urbana-Champaign, Urbana, IL 61801, USA; adpragya@illinois.edu; 2 Department of Agronomy, Iowa State University, Ames, IA 50011, USA; Mcnellie@iastate.edu; 3 Department of Horticultural Science, North Carolina State University, Mountain Horticultural Crops Research, and Extension Center, Mills River, Mills River, NC 28759, USA

**Keywords:** fruit quality, fruit shape, QTL analysis, *Solanum lycopersicum*

## Abstract

Tomato (*Solanum lycopersicum* L.) is the second most-consumed vegetable in the world. The market value and culinary purpose of tomato are often determined by fruit size and shape, which makes the genetic improvement of these traits a priority for tomato breeders. The main objective of the study was to detect quantitative trait loci (QTL) associated with the tomato fruit shape and size. The use of elite breeding materials in the genetic mapping studies will facilitate the detection of genetic loci of direct relevance to breeders. We performed QTL analysis in an intra-specific population of tomato developed from a cross between two elite breeding lines NC 30P × NC-22L-1(2008) consisting of 110 recombinant inbred lines (RIL). The precision software Tomato Analyzer (TA) was used to measure fruit morphology attributes associated with fruit shape and size traits. The RIL population was genotyped with the SolCAP 7720 SNP array. We identified novel QTL controlling elongated fruit shape on chromosome 10, explaining up to 24% of the phenotypic variance. This information will be useful in improving tomato fruit morphology traits.

## 1. Introduction

Tomato is the second most-consumed vegetable after potato in the United States, where the per capita consumption of the fresh market and processed tomato in 2017 were 20.3 lb (9.2 kg) and 73.3 lb (33.3 kg), respectively [1]. Tomato is consumed in various forms (fresh or processed) and is rich in vitamins A and C, as well as lycopene, which studies have suggested can decrease the odds of cancer and heart disease [2]. Tomato is also a model species for fleshy fruit development because of its genetic tractability and economic value. The origin of domestication for tomato is in the Andean region of South America and was spread to Europe as part of the Columbian exchange. During domestication and breeding, the tomato was selected for various fruit shapes and sizes. As a result, today, cultivated tomatoes have diverse shapes and sizes. The size of cultivated tomatoes ranges from small cherry size to medium, large, and extra-large fruited tomatoes. The shape of tomato can be classified into eight categories: flat, round, rectangular, ellipsoid, heart, long, obovoid, and oxheart [3].

The tomato fruit morphologies determine the culinary purposes (fresh consumption, sliced, diced, processed, or cooked) and market value of the fruits. Growers demand highly profitable tomatoes, which is correlated with the tomato sizes. The consumers tend to judge tomatoes first on visual appearance and taste second, although both traits are important [4]. The processing industry prefers rectangular and blocky tomatoes, as these shapes prevent the fruit from rolling from conveyor belts during mechanical harvesting [5]. The importance of tomato fruit shapes and sizes to consumers makes the genetic improvement of those traits a priority for tomato breeders. A better understanding of the genetic basis of fruit morphology will aid in their genetic improvement through breeding efforts.

Early molecular genetic studies in tomato focused on fruit size and shape because of their economic importance and ease of phenotyping. Several genes influencing tomato fruit size and shape have been identified, such as *SUN, OVATE,* cell number regulator *(CNR)/FW2.2, SlKLUH/FW3.2, FASCIATED* (*FAS*), and *LOCULE NUMBER* (*LC*) [6,7,8,9,10,11]. The *fas* mutation is present in most multilocular fresh-market tomatoes, whereas large and extra-large fruited tomatoes carry mutations in both the *fas* and *lc* loci. Epistatic interaction between *fas* and *lc* loci has been reported as a causal factor for the extremely large size of tomatoes mediated through high locule number as reviewed by van der Knaap et al. [12]. *SUN* confers uniform elongation to maintain bilateral symmetry as observed in most commercially grown tomatoes, heirloom, and oxheart tomatoes, whereas *OVATE* is responsible for the asymmetric elongation causing neck constriction or pear shape, as observed in ellipsoid and obovoid varieties of grape tomato [13]. A major fruit shape quantitative trait locus (QTL) *fs8.1* confers a blocky and slightly elongated shape in processing tomatoes and have arisen early in the tomato domestication process [14,15,16]. The two suppressors of *OVATE* gene *sov1* and *sov2* were also reported. The *sov1* was responsible for both obovoid and elongated shape, while *sov2* conferred elongated fruit shape [17].

These gene discoveries have explained the large proportions of diversity in the fruit morphology [3] and have enabled the breeding program to manipulate tomato fruit morphological traits to produce the fruits demanded by specific market classes. However, it is expected that there are undetected genes/loci controlling tomato fruit shapes and sizes because of the polygenic nature of such traits. Detection of additional genetic factors underlying tomato fruit shape and size is the first step in better understanding economically important fruit morphological traits. Most of the cloned fruit shape and size genes are homozygous in improved germplasm. Therefore, breeders need to detect loci of small effect within improved germplasm. The use of elite breeding materials will allow the detection of the minor allele effects, as most large effect alleles are homozygous in the population derived from closely related parents [17]. The use of intra-specific populations will, therefore, enable further understanding of the genetic mechanisms underlying fruit morphology by detecting novel alleles associated with variation in tomato [17]. Inter-specific populations, derived from crossing a wild relative to domesticated tomato, have traditionally been used to study fruit morphology due to the relative ease of detecting marker polymorphisms. Introgressing potentially advantageous alleles identified in inter-specific populations is difficult due to the confounding effect of different genetic backgrounds and deleterious linkage drag [18]. On the other hand, the use of elite breeding materials in QTL mapping study will facilitate the detection of QTL of direct relevance to breeders [19].

We performed QTL mapping for tomato fruit shape and size traits segregating in an intra-specific bi-parental fresh market tomato NC 10204 derived from the cross of two elite breeding lines. NC 10204 population was segregating for the ovate or pear-shaped trait and triangular fruit shape. We used the Solanaceae Coordinated Agricultural Project (SolCAP) 7720 SNP array [20] for genotyping and the precision phenotyping software Tomato Analyzer (TA) for phenotyping [21]. The effectiveness of TA and the SolCAP array in genetic studies of tomato have been previously demonstrated [17,22]. Few QTL mapping experiments in tomato have been conducted using intra-specific populations, and to our knowledge, there are limited studies on fruit shape and size using elite breeding material. 

Our objectives in this research were to detect QTL that influence fruit shape and size. We report novel QTL controlling elongated fruit shape characterized by fruit shape triangle. A better understanding of the loci influencing fruit shape and size will help breeders to improve these traits to the benefit of both growers and consumers.

## 2. Materials and Methods 

### 2.1. Population Development and Experimental Design

We developed the mapping population NC 10204 derived from the cross of the plum tomato breeding line NC 30P and the grape tomato breeding line NC 22L-1(2008) at Mountain Horticultural Crops Research and Extension Center (MHCREC), North Carolina State University, Mills River, NC. NC 30P has the crimson (*og^c^*) gene, imparting a dark-red fruit color, and also has enhanced lycopene content. NC 30P is a large and highly elongated fruits with 2–3 locules compared to NC 22L-1(228). Its average fruit weight is about 115–140 g per fruit. It was released as a plum breeding line for its superior horticultural traits from North Carolina State University tomato breeding program [23]. NC 22L-1(2008), an advanced grape breeding line derived from *S. pimpinellifolium* L3707, small elongated fruit with 2–3 locules. This is also dark-red and smooth. Its average fruit weight is about 15–20 g per fruit. The initial cross to develop an F_1_ hybrid was made in 2010. The F_1_ hybrid was self-pollinated to create a segregating F_2_ population, and F_2_ plants were individually harvested to create F_2:3_ families, which were further developed into F_5:6_ families using single seed descent.

The NC 10204 population was grown in MHCREC, Mills River, NC, during the 2014 (F_2:3_ generation) and 2017 (F_5:6_ generation) field seasons along with the parents in a random complete block design with two replications with one six-plant plot per line per replication. Replication was treated as a block effect. A total number of 129 lines and 110 lines were grown in the 2014 field season (F_2:3_ generation) and 2017 field season (F_5:6_ generation), respectively. The difference in the number of lines in two field seasons was due to the poor germination of the seeds. Individual plants were grown 45 cm apart within rows, 150 cm apart between rows, in a raised bed covered with plastic mulch with drip irrigation in the field. Plants were hand strung and sprayed according to the recommended schedule for fungicides and insecticides [24]. Seeds were germinated in 72 cell trays (56 × 28 cm^2^) in potting mix and grown for six weeks before hand transplantation into the field.

### 2.2. Phenotypic Analysis

Fruits were harvested from both field seasons and phenotyped for fruit size and shape using Tomato Analyzer (TA) version 3.0 [21]. A total of ten to twenty fruits per genotype per replication were selected for phenotyping in both the 2014 and 2017 field seasons. The selected fruits were cut proximal to distal (stem end to blossom end), and cut sections were scanned using a flatbed scanner (CanoScan 8800F, Canon USA Inc, Melville, NY, USA). The scanned fruit images were saved as jpeg files and imported into Tomato Analyzer 3.0 for automated phenotypic measurements. Images were manually adjusted as needed for corrections of borders and boundaries and analyzed as described by Rodríguez et al. [22]. The seven fruit size attributes—area (A), perimeter (P), width mid-height (WMH), maximum width (MW), height mid-width (HMW), maximum height (MH), curved height (CH) were recorded using TA. These size attributes represent the basic measurements in TA. Likewise, the seventeen fruit shape attributes–fruit shape index external I (FSI-ExtI), fruit shape index external II (FSI-ExtII), fruit shape index internal (FSI-Int), curved fruit shape index (CFSI), proximal fruit blockiness (PFB), distal fruit blockiness (DFB), fruit shape triangle (FST), ellipsoid (Ell), circular (Cir), rectangular (Rec), shoulder height (SH), proximal indentation area (PIA), obovoid (Ob), horizontal asymmetry obovoid (HOb), width widest position (WWP), eccentricity (Ecc), and eccentricity area index (EAI) were measured using TA. A full description of traits measured by TA can be found in the Tomato Analyzer Version 3 User Manual [21]. The measured data were exported from Tomato Analyzer for further analysis. 

Data analysis was performed in SAS software (version 9.4) [25]. Analysis of variance was performed in SAS using ‘PROC MIXED’ for both combined year data and individual year data. The broad-sense heritability was estimated for each environment (year) by calculating variance components using the ‘ASYCOV’ function in PROC MIXED in SAS. Phenotypic data were tested for normality using the Shapiro–Wilk test and traits with a *p*-value greater than 0.05 were considered normal. The data with non-normal residual distribution were transformed using square root transformation. The LSMEANS were calculated for individual year data. The final model to calculate LSMEANS by year included genotype as a fixed effect, and replication within a year, as random effects. The LSMEANS for each fruit shape and size attributes were exported for the QTL analysis. Pearson’s correlation coefficients were calculated for each attribute between two generations, and among various attributes within each generation using ‘PROC CORR.’ 

### 2.3. Linkage Map Construction and QTL Analysis

A total of 110 individuals (NC 10204) from both F_2:3_ and F_5:6_ generations were used for the QTL analysis. Genomic DNA was extracted from these individuals and parental lines using a modified cetyltrimethyl ammonium bromide (CTAB) method and quantified using a NanoDrop 2000 Spectrophotometer (Thermo Scientific, Wilmington, DE, USA) [26]. The F_2_, along with the parental lines, were genotyped using the SolCAP Illumina Infinium Assay. SNP genotypes were determined using GenomeStudio version 1.0 (Illumina Inc, San Diego, CA, USA). However, we lost several lines when we advanced the generation from F_2_ to F_5:6_ generation and ended up having only 110 lines for the field experiment.

The genetic map for NC 10204 was constructed using Joinmap 4.0 [27]. The regression mapping algorithm [28] was used to calculate marker order within each group. Kosambi mapping function was used for the estimation of map distances (cM) [29]. The physical map positions of the markers were obtained from Solanaceae Genome Network (SGN; http://solgenomics.net). 

Windows QTL Cartographer v 2.5 software was used for QTL analysis [30]. QTL analysis was conducted separately for each generation. The composite interval mapping (CIM) method using the default parameters (model 6) was used. A backward regression was used to perform the CIM analysis to enter or remove background markers from the model. The walking speed was set at one cM for the detection of QTL. The threshold LOD scores were calculated using 1000 permutations, and a significance level of 0.05 in QTL Cartographer and the loci with LOD value higher than the threshold were considered as QTL. QTL detected in both environments were reported as consistent QTL. The additive effect and the proportion of the observed phenotypic variation (R^2^) for each QTL were also obtained using this software. Dominance effects were not estimated as lines were mostly in the homozygous condition in F_5:6_ generation. QTL explaining more than 10% of the phenotypic variance was considered as major QTL [31,32]. Considering the bi-parental mapping population and the size of the mapping of the population used in this study, any QTL within 10 cM distance on the same chromosomes were regarded as a single QTL, and if detected in both environments were considered as a consistent QTL [33]. Only the consistent QTL were considered as true QTL and discussed.

## 3. Results

### 3.1. Phenotypic Variation

**Figure 1:**
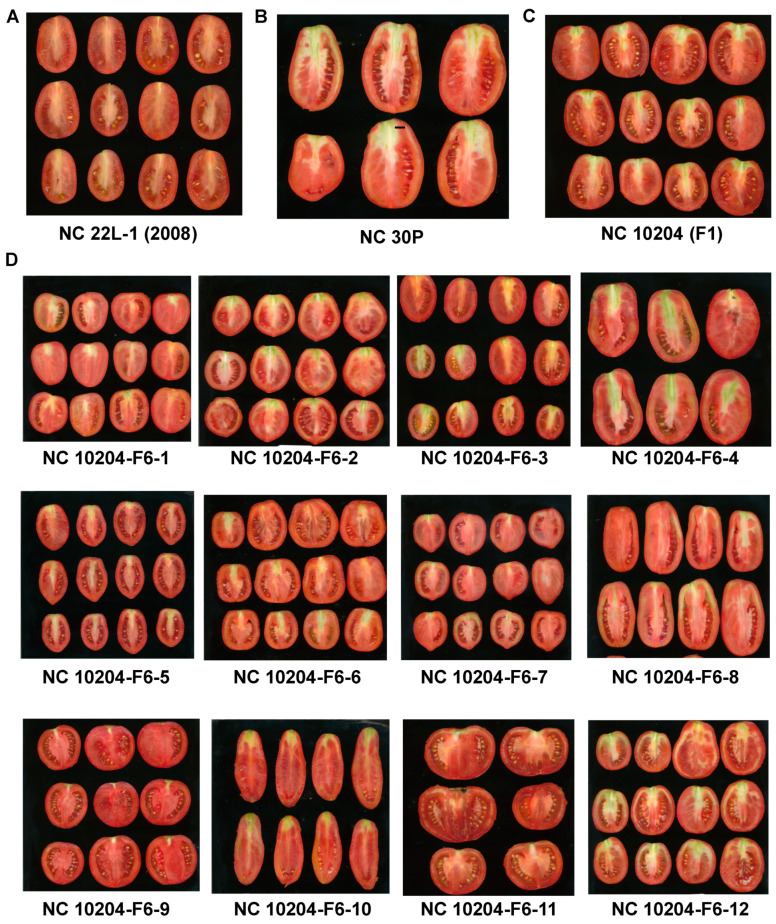
Fruit images cut from proximal to distal end of parental lines NC 22L-1(2008) (**A**), NC 30P (**B**), F_1_ hybrid (**C**), and the phenotypic segregation in the NC 10204 population (**D**).

**Figure 2:**
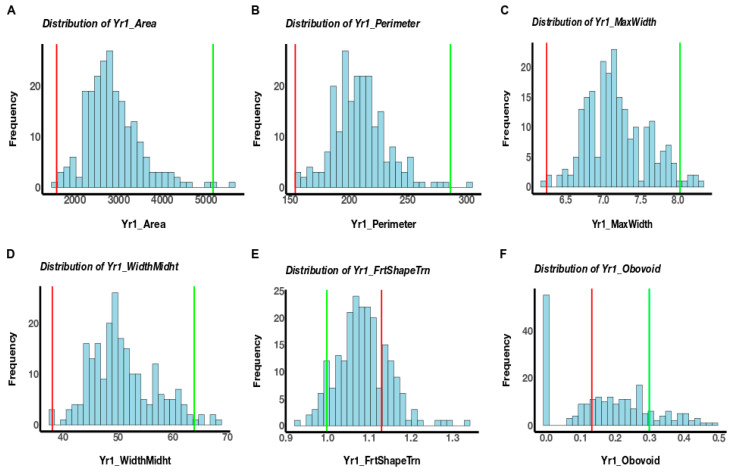
Phenotypic distribution of raw data for the area (**A**), perimeter (**B**), maximum width (**C**), width mid-height (**D**), fruit shape triangle (**E**), and obovoid (**F**) in the NC 10204 population in F_2:3_ generation. Lines were assessed using Tomato Analyzer software. Data were square root transformed for maximum width, fruit shape triangle, and obovoid. The red bar indicates LSMEAN of parent NC 22L-1(2008), and the green bar indicates LSMEAN of parent NC 30P.

**Figure 3:**
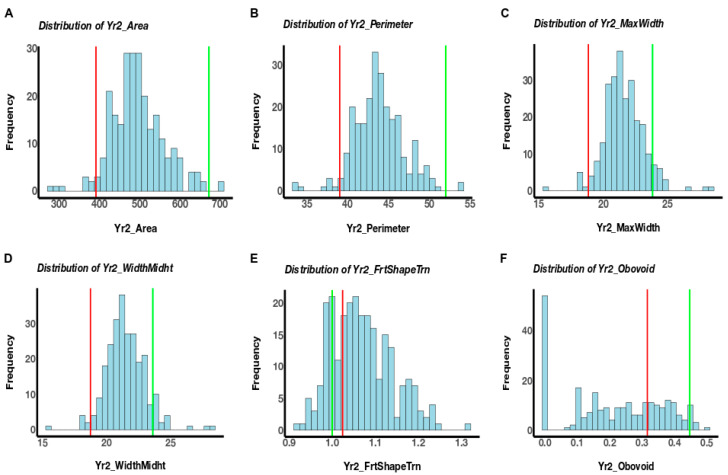
Phenotypic distribution of raw data for the area (**A**), perimeter (**B**), maximum width (**C**), width mid-height (**D**), fruit shape triangle (**E**), and obovoid (**F**) in the NC 10204 population in F5:6 generation. Data were square root transformed for all traits. Lines were assessed using Tomato Analyzer software. The red bar indicates LSMEAN of parent NC 22L-1(2008), and the green bar indicates LSMEAN of parent NC 30P.

The fruits of NC 30P were larger compared to that of NC 22L-1(2008), and the population segregated for various fruit morphologies (Figure 1). A total of twenty-four fruit morphology attributes (seven fruit size and seventeen fruit shape) were measured using TA in two generations, but only traits with the detected QTL will be discussed. The genotypic effect for these trait attributes measured was significant in both generations and the heritability estimates were moderate in the NC 10204 population, ranging from 0.56 to 0.77 (Table 1). The size attributes were higher in F_5:6_ generations (Table 1). All the attributes measured were significantly correlated at *p < 0.05* between F_2:3_ and F_5:6_ generations, except for ellipsoid (Table 1). Within each generation, significant correlations were detected amongst many traits, but only traits with detected QTL will be discussed. Among the fruit size traits, perimeter, area, width mid-height, and maximum width were significantly correlated (*p <* 0.001) in both generations (Table 2 and Table 3). Meanwhile, a significant negative correlation was observed between fruit shape triangle and obovoid (*p <* 0.001) in both generations (Table 2 and Table 3). We observed significant genotype X year interactions (Table 4). The phenotypic variation observed in the population in F_2:3,_ and F_5:6_ generations for these trait attributes are shown in Figure 2 and Figure 3.

### 3.2. Linkage Mapping and QTL Analysis

**Figure 4:**
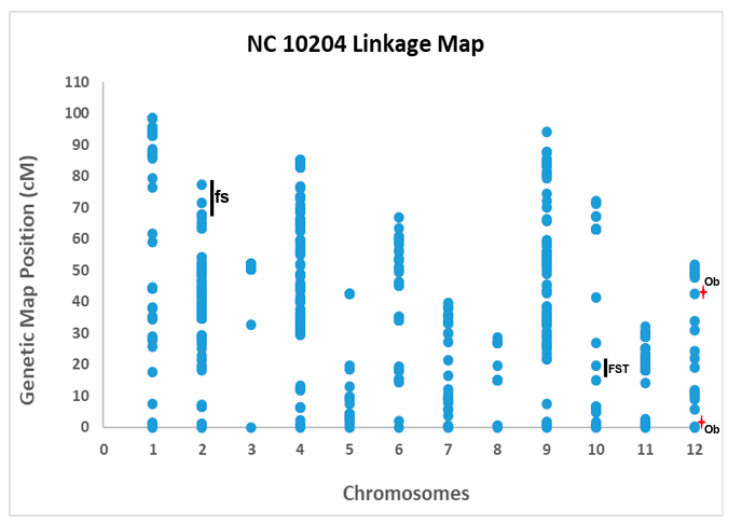
Linkage map of NC 10204. The x-axis represents chromosomes, and y-axis represents the genetic map position of the markers in each chromosome. The blue dots represent the markers in each chromosome. Black bars represent the QTL that were detected in two years in the same positions. The red star indicates the QTL that were detected in two years but in different genetic positions. ‘fs’ denotes fruit size traits that include area, perimeter, width mid-height, and maximum width, ‘FST’ denotes fruit shape triangle, and ‘Ob’ denotes obovoid.

Out of 7720 total SNP molecular markers that were used for genotyping, only 886 polymorphic SNP markers were included in the final linkage map construction of NC 10204 (Table 5). The final linkage map included 12 linkage groups. It spanned a total of 739.50 cM in the genetic distance with an average interval of 0.83 cM among 886 SNP polymorphic markers in NC 10204 linkage map (Table 5). The chromosome 1 had the longest length, and the chromosome 8 had the shortest length in the NC 10204 linkage map. The highest number of markers, i.e., 212, was observed in chromosome 4 in NC 10204 linkage map (Table 5). Large gaps between markers were observed on chromosome 3, 5, and 8 (Figure 4).

A total of five QTL were consistently detected in the same position across generations, four of which were associated with the fruit size attributes area, perimeter, maximum width (*MW*), and width mid-height (*WMH*), and one with fruit shape attribute fruit shape triangle (*FST*). All four QTL associated with the fruit size were co-localized on chromosome 2 (genetic map position 66.7 cM to 77.4 cM) with a LOD score value of 3.6 to 7.8 that explained up to 20% of the phenotypic variance (Figure 4, Table 6). The QTL associated with the *FST* was observed on chromosome 10 (genetic map position between 16.07 cM to 22.7 cM) with a LOD score value up to 5.4 (Table 6). For the obovoid (*Ob*) trait, we observed QTL on the same chromosome (12), but on different positions in different generations with a LOD score value greater than 4, explaining up to 34% of the phenotypic variance (Figure 4, Table 6). The positive additive effects were associated with the parent NC 30P, and negative additive effects were associated with the parent NC 22L-1(2008) (Table 6).

## 4. Discussion

Fruit shape and size are polygenically inherited with effects ranging from small to large [34]. Genetic studies to elucidate molecular mechanisms of fruit morphology traits in tomato have historically employed inter-specific populations derived from crossing cultivated and wild tomato species to obtain a sufficient number of polymorphic markers [17]. However, introgressing QTL from wild or unimproved genotypes into elite lines often suffers from linkage drag and requires extensive backcrossing [19]. Recently, intra-specific mapping populations have been used to study the genetic control of tomato fruit shape using the SolCAP SNP array [17,22]. 

In this study, we identified five QTL in both the F_2:3_ and F_5:6_ generations controlling fruit shape and size traits using an intra-specific bi-parental mapping population derived from the elite tomato breeding lines NC 30P and NC 22L-1(2008). QTL for two attributes of fruit shape traits—fruit shape triangle and obovoid—were mapped in this study. Fruit shape triangle QTL (*tri)* controlling elongated fruit shape was mapped to a 7 cM region on chromosome 10 that explained 9–24% of the total phenotypic variance. Previously, QTL for *tri* has been mapped to chromosome 7 in three populations, and on chromosome 3 in one population [35]. The *tri* QTL was also detected on chromosomes 1, 2, 3, and 11 in three populations in another study [13]. In our study, the QTL for fruit shape triangle did not overlap with major fruit shape loci on chromosome 7 and other previously detected QTL, indicating a different locus on chromosome 10 independently controls the fruit shape triangle in NC 10204. To the extent of our knowledge, the fruit shape triangle QTL has not been reported on chromosome 10, indicating that this is a novel QTL.

One of the observations made in the present experiment was that there was a significant genotype by year interaction effect and a large difference in contribution rates between the two generations, even in the same trait (Table 6). This might be due to the level of homozygosity in the population. The F_2:3_ has a higher level of heterozygosity, whereas F_5:6_ is almost at working homozygous level. This may be reflected in the phenotypic level, which eventually affects the QTL analysis and allele contribution rate.

Four fruit size attributes—area, perimeter, maximum width, and width mid-height—were co-localized to a region between 66.7 and 77.4 cM in the genetic map on chromosome 2 in both generations that explained up to 20% of phenotypic variance. The significant correlations between these four attributes suggest that a single QTL might be controlling all these four traits. Three cloned genes that influence fruit size are located on chromosome 2 (*ovate, fw2.2,* and *lcn2.1*), as well as at least three other putative QTL [36]. The closest markers associated with the detected QTL for fruit size in our study were located on chromosome 2 at physical map positions of 44,069,445 to 47,948,927 bp), which is close to the position of *Fw2.2/CNR* (Solyc02g090730) (52,252,556 to 52,253,347 bp) according to the gene information in Sol Genomics Network (SGN) [37]. This suggests that NC 10204 is segregating for major fruit size loci. 

The obovoid QTL responsible for the pear-shaped tomato was mapped to chromosome 12, but on different genetic positions in different generations, despite the significant correlations (r = 0.5, *p <* 0.001) between two generations. The closest markers associated with the QTL in different generations were located at different positions (~62–78 Mb in F_5:6_ and ~40 to 50 Mb in F_2:3_) on chromosome 12 in the physical map too. In addition, the loci at two different positions were associated with two different parents. This suggests that there might be two different QTL controlling obovoid fruit shape attributes in NC 10204 contributed from both parents. This needs further verification. However, it is worth discussing the QTL associated with the obovoid in our study, as they were detected on chromosome 12 in both generations. Previously, the major fruit shape genes *SUN* and *OVATE* responsible for fruit elongation were located on chromosomes 7 and 2, respectively [9,11], while the two suppressors of ovate (sov1 and sov2) controlling obovoid and elongated shape were mapped to chromosomes 10 and 11 [17]. Huang et al. [38] detected one of the *OVATE*-like genes (*SlOFP16*) on the long arm of chromosome 8. Chusreeaeom et al. [39] also detected the *Slelf1* mutant candidate gene controlling elongated fruit shape on the long arm of chromosome 8, but at a different position than that of *SlOFP16*. This suggests that the QTL associated with the obovoid in our study were detected in genomic regions not identified by prior studies and might be interesting, although they were detected in different positions in two generations. 

This study identified a novel QTL controlling fruit shape triangle on chromosomes 10 and discovered the possibility of two novel QTL controlling obovoid on chromomse 12. At the same time, this study also identified the previously detected genetic loci controlling fruit size in the inter-specific tomato population. This information will be useful to resolve fruit shape variation in cultivated tomato species fully.

## Figures and Tables

**Table 1:** Descriptive statistics for fruit morphological attributes measured by Tomato Analyzer (TA) in two generations (F_2:3_ and F_5:6_), significance test of genotypic effects on the attributes measured, normality test of residuals, broad-sense heritability (H^2^) and the correlation coefficients between generations in the intra-specific population NC 10204 of tomato.

Category of Trait	Trait	F_2:3_(Year 2014)							F_2:3_(Year 2014)							Correlation between Generations ^c^
		Mean	Std. Dev	Min.	Max.	Geno Effect ^a^	Shapiro-Wilk Normality Test of Residuals ^b^	H^2^	Mean	Std. Dev	Min.	Max.	Geno Effect	Shapiro-Wilk Normality Test of Residuals	H^2^	
Fruit Size	**A**	2836	549.2	1640	5095	<0.0001	0.4236	0.70	247,274	49,705	130,233	416,683	<0.0001	<2.2 × 10^16 nn^	0.61	0.25 *
	**P**	207.8	20.38	155	281.9	<0.0001	0.3971	0.69	1929	206.2	1403	2566	<0.0001	<2.2 × 10^16 nn^	0.64	0.25 *
	**WMH**	50.62	5.39	40.8	66.45	<0.0001	0.06062 ^nn^	0.73	460.9	50.34	300.3	599.83	<0.0001	<2.2 × 10^16 nn^	0.64	0.24 *
	**MW**	51.46	5.4	42.3	67.26	<0.0001	0.04489	0.73	466.2	50.35	304.1	601.75	<0.0001	<2.2 × 10^16 nn^	0.73	0.24 *
Fruit Shape	**FST**	1.18	0.13	0.87	1.77	<0.0001	0.00201 ^nn^	0.56	1.15	0.14	0.87	1.51	<0.0001	0.001386 ^nn^	0.67	0.52 ***
	**Ob**	0.05	0.05	0	0.24	<0.0001	4.77 × 10^6 nn^	0.64	0.07	0.06	0	0.23	<0.0001	5.18 × 10^11 nn^	0.77	0.48 ***

Note: ^a^ The *p*-value determining the significance of genotypic effects. ^b^ The *p*-value of the Shapiro–Wilk normality test of residuals. A *p*-value below 0.05 indicates that the data significantly deviate from a normal distribution. Here, ‘nn’ represents non-normal distribution of the residuals. The non-normal data were square root transformed for further analysis. ^c^ *** denotes *p*-value < 0.001, * *p*-value < 0.05. Trait: A = area, P = perimeter, WMH = width mid-height, MW = maximum width, FST = fruit shape triangle, Ob = obovoid.

**Table 2:** Pearson’s correlation coefficient among fruit morphology traits in the intra-specific tomato population NC 10204 in F_2:3_ generation.

Variable	P	A	WMH	MW	FST	Ob
P	1					
A	0.99 ***^a^	1				
WMH	0.8 ***	0.86 ***	1			
MW	0.81 ***	0.86 ***	0.99 ***	1		
FST	−0.19 *	−0.18 *	ns ^b^	ns	1	
Ob	0.22 *	ns	−0.18 *	ns	−0.77 ***	1

^a^ *** denotes *p*-value < 0.001, * *p*-value < 0.05. ^b^ ns represents non-significant. Trait: P = perimeter, A = area, WMH = width mid-height, MW = maximum width, FST = fruit shape triangle, and Ob = obovoid.

**Table 3:** Pearson’s correlation coefficient among fruit morphology traits in the intra-specific tomato population NC 10204 in F_5:6_ generation.

Variable	P	A	WMH	MW	FST	Ob
P	1					
A	0.98 ***^a^	1				
WMH	0.70 ***	0.79 ***	1			
MW	0.73 ***	0.81 ***	0.99 ***^b^	1		
FST	−0.2 *	ns	ns	ns	1	
Ob	0.33 ***	0.23 *	ns	ns	−0.83 ***	1

^a^ *** denotes *p*-value < 0.001, * *p*-value < 0.05. ^b^ ns represents non-significant. Trait: P = perimeter, A = area, WMH = width mid-height, MW = maximum width, FST = fruit shape triangle, and Ob = obovoid.

**Table 4:** Source of variation for the phenotypes observed for the traits with detected quantitative trait loci (QTL).

Source	Perimeter		Area		Width Mid Height		Maximum Width		Fruit Shape Triangle		Obovoid	
	F Value	Pr > F	F Value	Pr > F	F Value	Pr > F	F Value	Pr > F	F Value	Pr > F	F Value	Pr > F
**Genotype**	1.5	0.0213 *	1.55	0.0148 *	1.45	0.0316 *	1.35	0.0648	2.59	<0.0001 ***	2.52	<0.0001 ***
**Year**	607.3	0.0002 ***	2579.94	<0.0001 ***	2055.84	<0.0001 ***	2508.6	<0.0001 ***	2.48	0.1256	2.44	0.151
Genotype * Year	3.31	<0.0001 ***	4.94	<0.0001 ***	5.31	<0.0001 ***	3.59	<0.0001 ***	2.01	<0.0001 ***	2.39	<0.0001 ***
**Replication (Year)**	17.99	<0.0001 ***	0.44	0.6433	3.27	0.0401 *	23.25	<0.0001 ***	0.37	0.6897	1.94	0.1457

*** denotes *p*-value < 0.001, * denotes *p*-value < 0.05.

**Table 5:** Summary of the linkage map of NC 10204 showing 12 chromosomes along with the number of markers per chromosome and the length of each chromosome.

Chromosome	Markers	Length (cM)
1	58	98.6
2	101	77.4
3	60	52.1
4	212	85.3
5	30	42.6
6	60	66.8
7	36	39.6
8	15	28.8
9	138	94.1
10	31	72.1
11	112	30.1
12	33	51.9
Total	886	739.4

**Table 6:** Summary of QTL information detected across different generations in NC10204 population of tomato controlling fruit size and shape. The table presents the position of the QTL in genetic map and physical map, flanking SNP molecular markers, LOD score value and phenotypic variation explained by each QTL (R^2^-value), additive effect, and if the QTL has been previously identified or novel. Y1 indicates the year 2014 (F_2:3_ generation), and Y2 indicates the year 2017 (F_5:6_ generation). The positive additive effects were associated with the parent NC 30P, and negative additive effects were associated with the parent NC 22L-1(2008). The physical map position of the markers was obtained from the Solanaceae Genome Network (SGN; http://solgenomics.net).

Category of Traits	Traits	Chr.	Closest Markers	Genetic Map Position (cM)	Physical Map Position (bp)	Threshold LOD	LOD Score	Additive Value	R2 (%)	Nature of QTL
**Fruit Size**	Yr1_P	2	solcap_snp_sl_21867	77.44	47,948,927	3.7	6.9	−7.76	6.9	^a^ consistent; confirmatory
	Yr2_P	2	solcap_snp_sl_50066	67.7	44,783,686	3.6	4.7	−2.17	17.2	consistent; confirmatory
	Yr1_A	2	solcap_snp_sl_21867	75.44	47,948,927	3.8	7.8	−228.27	8.3	consistent; confirmatory
	Yr2_A	2	solcap_snp_sl_42324	66.73	44,069,445	3.7	5.4	−50.58	20.0	consistent; confirmatory
	Yr1_WMH	2	solcap_snp_sl_21867	77.44	47,948,927	3.9	7.7	−2.23	8.8	consistent; confirmatory
	Yr2_WMH	2	solcap_snp_sl_21867	77.44	47,948,927	3.7	3.7	−0.72	15.8	consistent; confirmatory
	Yr1_MW	2	solcap_snp_sl_21867	72.44	47,948,927	3.6	6.9	−0.16	8.3	consistent; confirmatory
	Yr2_MW	2	solcap_snp_sl_21867	76.44	47,948,927	3.6	3.6	−0.73	15.6	consistent; confirmatory
**Fruit Shape**	Yr1_FST	10	solcap_snp_sl_9598, solcap_snp_sl_16517	22.77	4,260,136;57,327,585	3.7	4.0	0.03	10.2	consistent; novel
	Yr2_FST	10	solcap_snp_sl_34373, solcap_snp_sl_9598	16.07	3,991,802;4,260,136	3.6	5.4	0.05	24.2	consistent; novel
	Yr1_Ob	12	solcap_snp_sl_1573, solcap_snp_sl_58869	1.26	4,038,812;5,029,856	3.7	4.9	−0.10	34.0	^b^ not consistent; putative QTL; might be novel
	Yr2_Ob	12	solcap_snp_sl_24755, solcap_snp_sl_31628	43.48	7,801,435;64,210,355	3.7	4.2	0.06	7.1	not consistent; putative QTL; might be novel

Trait: P = perimeter, A = area, WMH = width mid-height, MW = maximum width, FST = fruit shape triangle, Ob = obovoid. **^a^** QTL that were detected on the close genetic positions (<10 cM) across different generations. **^b^** QTL that were detected on the different genetic positions (>10 cM) across different generations.

## References

[1] AMRC (2018). Tomatoes.

[2] Merk H.L., Ashrafi H., Foolad M.R. (2012). Selective genotyping to identify late blight resistance genes in an accession of the tomato wild species *Solanum pimpinellifolium*. Euphytica.

[3] Rodriguez G.R., Munos S., Anderson C., Sim S.C., Michel A., Causse M., Gardener B.B.M., Francis D., van der Knaap E. (2011). Distribution of SUN, OVATE, LC, and FAS in the tomato germplasm and the relationship to fruit shape diversity. Plant Physiol..

[4] Barrett D.M., Beaulieu J.C., Shewfelt R. (2010). Color, flavor, texture, and nutritional quality of fresh-cut fruits and vegetables: Desirable levels, instrumental and sensory measurement, and the effects of processing. Crit. Rev. Food Sci. Nutr..

[5] Visa S., Cao C.X., Gardener B.M., van der Knaap E. (2014). Modeling of tomato fruits into nine shape categories using elliptic fourier shape modeling and Bayesian classification of contour morphometric data. Euphytica.

[6] Chakrabarti M., Zhang N., Sauvage C., Munos S., Blanca J., Canizares J., Diez M.J., Schneider R., Mazourek M., McClead J. (2013). A cytochrome P450 regulates a domestication trait in cultivated tomato. Proc. Natl. Acad. Sci. USA.

[7] Cong B., Barrero L.S., Tanksley S.D. (2008). Regulatory change in YABBY-like transcription factor led to evolution of extreme fruit size during tomato domestication. Nat. Genet..

[8] Frary A., Nesbitt T.C., Grandillo S., van der Knaap E., Cong B., Liu J.P., Meller J., Elber R., Alpert K.B., Tanksley S.D. (2000). fw2.2: A quantitative trait locus key to the evolution of tomato fruit size. Science.

[9] Liu J.P., Van Eck J., Cong B., Tanksley S.D. (2002). A new class of regulatory genes underlying the cause of pear-shaped tomato fruit. Proc. Natl. Acad. Sci. USA.

[10] Munos S., Ranc N., Botton E., Berard A., Rolland S., Duffe P., Carretero Y., Le Paslier M.C., Delalande C., Bouzayen M. (2011). Increase in tomato locule number is controlled by two Single Nucleotide Polymorphisms located near WUSCHEL. Plant Physiol..

[11] Xiao H., Jiang N., Schaffner E., Stockinger E.J., van der Knaap E. (2008). A retrotransposon-mediated gene duplication underlies morphological variation of tomato fruit. Science.

[12] Van der Knaap E., Chakrabarti M., Chu Y.H., Clevenger J.P., Illa-Berenguer E., Huang Z.J., Keyhaninejad N., Mu Q., Sun L., Wang Y.P. (2014). What lies beyond the eye: The molecular mechanisms regulating tomato fruit weight and shape. Front. Plant Sci..

[13] Gonzalo M.J., van der Knaap E. (2008). A comparative analysis into the genetic bases of morphology in tomato varieties exhibiting elongated fruit shape. Theor. Appl. Genet..

[14] Grandilio S., Ku H.M., Tanksley S.D. (1996). Characterization of fs8.1, a major QTL influencing fruit shape in tomato. Mol. Breed..

[15] Ku H.M., Grandillo S., Tanksley S.D. (2000). fs8.1, a major QTL, sets the pattern of tomato carpel shape well before anthesis. Theor. Appl. Genet..

[16] Clevenger J. (2012). Metabolic and Genomic Analysis of Elongated Fruit Shape in Tomato (*Solanum lycopersicum*). Master’s Thesis.

[17] Rodríguez G.R., Kim H.J., van der Knaap E. (2013). Mapping of two suppressors of OVATE (sov) loci in tomato. Heredity.

[18] Lecomte L., Duffe P., Buret M., Servin B., Hospital F., Causse M. (2004). Marker-assisted introgression of five QTLs controlling fruit quality traits into three tomato lines revealed interactions between QTLs and genetic backgrounds. Theor. Appl. Genet..

[19] Wurschum T. (2012). Mapping QTL for agronomic traits in breeding populations. Theor. Appl. Genet..

[20] Sim S.-C., Durstewitz G., Plieske J., Wieseke R., Ganal M.W., Van Deynze A., Hamilton J.P., Buell C.R., Causse M., Wijeratne S. (2012). Development of a large SNP genotyping array and generation of high-density genetic maps in tomato. PLoS ONE.

[21] Brewer M.T., Lang L.X., Fujimura K., Dujmovic N., Gray S., van der Knaap E. (2006). Development of a controlled vocabulary and software application to analyze fruit shape variation in tomato and other plant species. Plant Physiol..

[22] Rodríguez G., Moyseenko J., Robbins M., Morejón N., Francis D., van der Knaap E. (2010). Tomato Analyzer: A useful software application to collect accurate and detailed morphological and colorimetric data from two-dimensional objects. J. Vis. Exp..

[23] Gardner R.G., Panthee D.R. (2010). ‘Plum Regal’ Fresh-market plum tomato hybrid and its parents, NC 25P and NC 30P. HortScience.

[24] Ivors K. (2010). Commercial Production of Staked Tomatoes in the Southeast.

[25] (2012). The SAS System, Version 9.4 for Windows.

[26] Kabelka E., Franchino B., Francis D.M. (2002). Two loci from *Lycopersicon hirsutum* LA407 confer resistance to strains of *Clavibacter michiganensis* subsp michiganensis. Phytopathology.

[27] Van Ooijen J.W. (2006). Joinmap 4.0, Software for the Calculation of Genetic Linkage Maps in Experimental Populations.

[28] Stam P. (1993). Construction of integrated genetic linkage maps by means of a new computer package: Join Map. Plant J..

[29] Kosambi D.D. (1943). The estimation of map distances from recombination values. Ann. Eugen..

[30] (2012). Windows QTL Cartographer, V2.5.

[31] Panthee D.R., Piotrowski A., Ibrahem R. (2017). Mapping Quantitative Trait Loci (QTL) for Resistance to Late Blight in Tomato. Int. J. Mol. Sci..

[32] Panthee D.R., Pantalone V.R., Sams C.E., Saxton A.M., West D.R., Orf J.H., Killam A.S. (2006). Quantitative trait loci controlling sulfur containing amino acids, methionine and cysteine, in soybean seeds. Theor. Appl. Genet..

[33] Pascual L., Desplat N., Huang B.E., Desgroux A., Bruguier L., Bouchet J.P., Le Q.H., Chauchard B., Verschave P., Causse M. (2015). Potential of a tomato MAGIC population to decipher the genetic control of quantitative traits and detect causal variants in the resequencing era. Plant Biotechnol. J..

[34] Ben Chaim A., Borovsky Y., Rao G., Gur A., Zamir D., Paran I. (2006). Comparative QTL mapping of fruit size and shape in tomato and pepper. Isr. J. Plant Sci..

[35] Brewer M.T., Moyseenko J.B., Monforte A.J., van der Knaap E. (2007). Morphological variation in tomato: A comprehensive study of quantitative trait loci controlling fruit shape and development. J. Exp. Bot..

[36] Lin T., Zhu G.T., Zhang J.H., Xu X.Y., Yu Q.H., Zheng Z., Zhang Z.H., Lun Y.Y., Li S., Wang X.X. (2014). Genomic analyses provide insights into the history of tomato breeding. Nat. Genet..

[37] Fernandez-Pozo N., Menda N., Edwards J.D., Saha S., Tecle I.Y., Strickler S.R., Bombarely A., Fisher-York T., Pujar A., Foerster H. (2015). The Sol Genomics Network (SGN)-from genotype to phenotype to breeding. Nucleic Acids Res..

[38] Huang Z.J., Van Houten J., Gonzalez G., Xiao H., van der Knaap E. (2013). Genome-wide identification, phylogeny and expression analysis of SUN, OFP and YABBY gene family in tomato. Mol. Genet. Genom..

[39] Chusreeaeom K., Ariizumi T., Asamizu E., Okabe Y., Shirasawa K., Ezura H. (2014). A novel tomato mutant, *Solanum lycopersicum* elongated fruit1 (Slelf1), exhibits an elongated fruit shape caused by increased cell layers in the proximal region of the ovary. Mol. Genet. Genom..

